# Female Pacific walruses (*Odobenus rosmarus divergens*) show greater partitioning of sea ice organic carbon than males: Evidence from ice algae trophic markers

**DOI:** 10.1371/journal.pone.0255686

**Published:** 2021-08-19

**Authors:** Chelsea W. Koch, Lee W. Cooper, Ryan J. Woodland, Jacqueline M. Grebmeier, Karen E. Frey, Raphaela Stimmelmayr, Cédric Magen, Thomas A. Brown

**Affiliations:** 1 University of Maryland Center for Environmental Science, Solomons, Maryland, United States of America; 2 Graduate School of Geography, Clark University, Worcester, Massachusetts, United States of America; 3 North Slope Borough Department of Wildlife Management, Utqiaġvik, Alaska, United States of America; 4 Institute of Arctic Biology, University of Alaska, Fairbanks, Alaska, United States of America; 5 Scottish Association for Marine Science, Oban, Scotland, United Kingdom; Universita del Salento, ITALY

## Abstract

The expected reduction of ice algae with declining sea ice may prove to be detrimental to the Pacific Arctic ecosystem. Benthic organisms that rely on sea ice organic carbon (iPOC) sustain benthic predators such as the Pacific walrus (*Odobenus rosmarus divergens*). The ability to track the trophic transfer of iPOC is critical to understanding its value in the food web, but prior methods have lacked the required source specificity. We analyzed the H-Print index, based on biomarkers of ice algae versus phytoplankton contributions to organic carbon in marine predators, in Pacific walrus livers collected in 2012, 2014 and 2016 from the Northern Bering Sea (NBS) and Chukchi Sea. We paired these measurements with stable nitrogen isotopes (*δ*^15^N) to estimate trophic position. We observed differences in the contribution of iPOC in Pacific walrus diet between regions, sexes, and age classes. Specifically, the contribution of iPOC to the diet of Pacific walruses was higher in the Chukchi Sea (52%) compared to the NBS (30%). This regional difference is consistent with longer annual sea ice persistence in the Chukchi Sea. Within the NBS, the contribution of iPOC to walrus spring diet was higher in females (~45%) compared to males (~30%) for each year (p < 0.001), likely due to specific foraging behavior of females to support energetic demands associated with pregnancy and lactation. Within the Chukchi Sea, the iPOC contribution was similar between males and females, yet higher in juveniles than in adults. Despite differences in the origin of organic carbon fueling the system (sea ice versus pelagic derived carbon), the trophic position of adult female Pacific walruses was similar between the NBS and Chukchi Sea (3.2 and 3.5, respectively), supporting similar diets (i.e. clams). Given the higher quality of organic carbon from ice algae, the retreat of seasonal sea ice in recent decades may create an additional vulnerability for female and juvenile Pacific walruses and should be considered in management of the species.

## Introduction

Primary production in the Pacific Arctic region is partitioned between sympagic (sea ice) and pelagic (open water) sources [1]. On the continental shelf there is a tight sympagic-pelagic-benthic coupling resulting in high benthic biomass [2], which supports a variety of specialized benthic predators such as the Pacific walrus (*Odobenus rosmarus divergens*) [3,4]. This ecosystem is undergoing a shift from a benthic-dominated system to a more pelagic-based one due in part to changes in ice algal and phytoplankton production [5]. Ice algae are considered to be an important food source for benthic organisms owing to the early timing of the blooms, which are rapidly exported to the benthos [6]. However, while ice algae are likely decreasing in some locations, phytoplankton blooms are increasing in response to sea ice declines [7,8]. It is unclear how this shift will impact food webs in the Pacific Arctic [9,10]. We selected the Pacific walrus (*O*. *r*. *divergens*) to investigate how sea ice primary production is presently being incorporated in the upper trophic positions of this benthic-dominated system. Declining sea ice not only affects foraging behavior and access to foraging grounds of the Pacific walrus [11,12], but it is also likely to impact the quality of their diet owing to shifting carbon sources. Monitoring sea ice organic carbon contributions to Pacific walrus diets has the potential to provide another informative tool in assessing one of several ways in which climate change will impact this species.

The Pacific walrus serves as an interesting test case for several reasons including their benthic based diet, geographic range relative to sea ice areal coverage, the migratory behavior of this species, and their general reliance on sea ice as a physical habitat. Pacific walruses are generalists that consume a wide range of prey items [13–15]. They are benthic specialist predators with the ability to forage 30 cm deep into sediments [16]. Bivalves are their preferred prey item but their diet can also include large contributions of gastropods and polychaetes in the Chukchi and Bering seas [13,17]. Most of what is known about the Pacific walrus diet has come from fresh stomach content studies, acknowledging that some prey items are underrepresented with this method [13,18]. Uncertainties remain regarding the breadth of their diets and the flexibility that exists to adapt and partition resources with other benthic predators in the region [19]. With expanding northward ranges of boreal species, there may also be increasing competition for benthic resources, as was recently observed for the Atlantic walrus (*O*. *r*. *rosmarus*) population [20].

**Fig 1:**
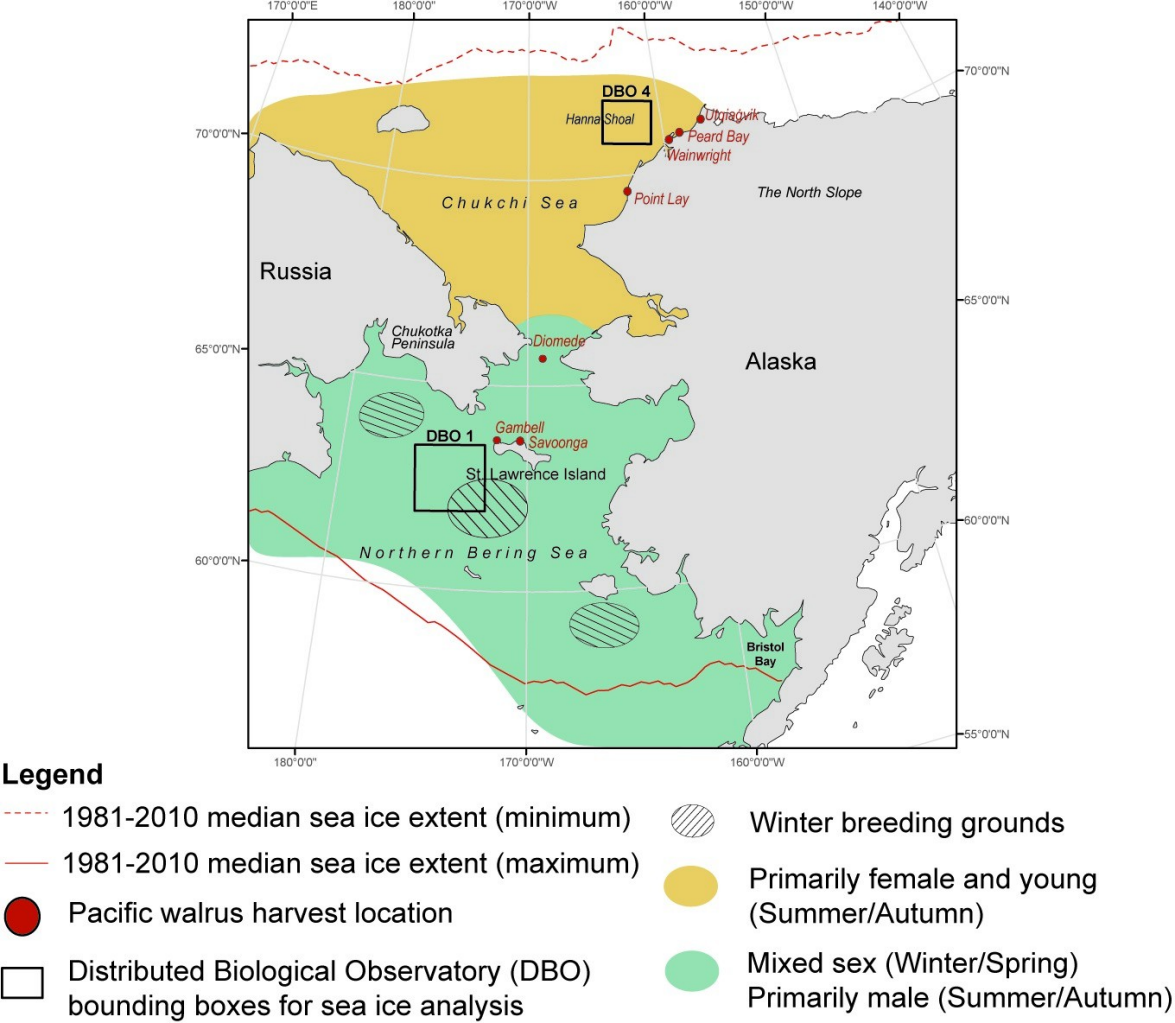
The Pacific walrus geographical range and sample harvest locations. Pacific walrus liver tissues were collected by subsistence hunters from several communities in the Bering Strait and from the North Slope of Alaska. The range of the Pacific walrus spans the northern Bering and Chukchi Seas, with sex-segregated distributions throughout the year (green/yellow shading). The Distributed Biological Observatory regions 1 and 4 (boundaries denoted by the black boxes; Grebmeier et al. [4]) encompass prominent Pacific walrus breeding and foraging grounds, which were used for extracting satellite-derived sea ice measurements and nitrogen stable isotope data for primary consumers from previous studies for the relative trophic position analysis. Pacific walrus range data was modified from Garlich-Miller et al. [22] and Smith [23]. Open-source coastline data was retrieved from Natural Earth (https://www.naturalearthdata.com/).

As an ice-obligate species [21], the geographic range of the Pacific walrus overlaps with the seasonal ice coverage in the region (Fig 1) [22,23]. However, they are limited to the shallow waters over the continental shelf where they can effectively forage [18]. This adaptation has proven to be an issue in recent years because the ice edge has consistently retreated off of the shelf into the deeper waters of the Arctic Ocean, limiting an important platform for foraging walruses in the late summer [11,24]. As a result of the northerly ice edge location, walruses are increasingly using coastal haul-outs along the Alaskan coast and Chukotka peninsula where food resources are scarce [11]. The lack of sea ice inhibits their ability to forage further offshore where benthic biomass is substantially higher than these nearshore locations [11]. The ice-obligate classification of the Pacific walrus is also because sea ice provides a necessary platform for female walruses to give birth and nurse their young [18]. Therefore, females and dependent calves are particularly reliant on the presence of sea ice; they migrate annually with the ice edge into the Chukchi Sea where they remain (when possible) through the fall until the ice begins to re-form [18]. The breeding season occurs in the winter and early spring when the females return to the Bering Sea. Contrary to the apparent sea ice requirements for females, male haul-out locations are prominent throughout the year along the western ice-free portions of the Bearing Sea coast in Russia and in Bristol Bay in the southeast Bering Sea in open water conditions [25].

South of St. Lawrence Island, in the NBS, is an important wintering ground and breeding area for the Pacific walrus population [25,26]. The area is the site of an annually recurrent winter polynya (an area of open water in otherwise ice-covered waters) that generates cold bottom water during sea ice formation and elevated nutrient supply, leading to high benthic biomass [27]. The polynya formation results in an ideal walrus foraging location [26]. Tellinid bivalves (i.e. *Macoma*) are historically prevalent in this region and areas of high biomass were determined to be significantly correlated with Pacific walrus foraging site selection [26]. However, this region is changing in response to a warming climate and the bivalve communities have been shifting northwards over the past few decades [28]. The once dominant Tellinid and Nuculanid bivalves in the southern part of the region have been replaced by polychaete worms [29], possibly suggesting changes at the base of the food web. As the sea ice retreats earlier in the year, or never reaches previously covered areas (e.g. 2018 and 2019, see Frey et al. and Clement Kinney, this issue), an early spring ice-edge bloom does not occur [30,31]. Studies have hypothesized that a retreating winter ice edge and shifting benthic populations could lead to a northward shift in the walrus wintering grounds and changes in their prey selection [26,32]. The NBS is transitioning to a pelagic dominated system with a declining benthic standing stock that will have consequences for marine mammals, particularly the benthic-feeding Pacific walrus [29,33].

Walruses segregate into distinct groups where most adult males remain in Bristol Bay and the Russian Bering Sea coast while females, dependent calves and young walruses follow the ice edge north into the Chukchi Sea each spring where they stay until autumn [18,25]. Declining sea ice has consequences for these summer and autumn foraging grounds in the Chukchi Sea [11,12,25]. The southeastern flanks of Hanna Shoal in the northeast Chukchi Sea have sufficiently high bivalve biomass to support the caloric density requirements to sustain walrus foraging activities [34–36]. However, access to Hanna Shoal is largely dependent on the presence of sea ice given its distant offshore location, which allows the walruses to haul out on ice to rest and remain offshore [12,32]. Without sufficient sea ice coverage on the shelf, these walruses are forced to seek out coastal haul-outs, either utilizing scarce nearshore resources or traveling much greater distances to reach their preferred foraging grounds [11,12,32]. The benthic communities closer to the Alaskan shore are influenced by the Alaska Coastal Current [37], which is warmer, fresher and nutrient-poor compared to the Bering Sea water that flows towards Hanna Shoal in the summer [38,39]. The nutrient-rich waters of Hanna Shoal, coupled with prolonged presence of sea ice [40], allow for sustained ice algae blooms that likely fuels the high benthic biomass there [4,41]. Ice algae appear to be a more prominent food source for the benthic communities of the Chukchi Sea compared to those of the NBS [42] and may be a source of more lipid-rich prey for walruses.

We measured highly branched isoprenoids (HBIs) lipids, which are biomarkers produced by diatoms with well-defined sea ice and phytoplankton sources [43–45] and the H-Print index [14] to track the trophic transfer of these two carbon sources. One HBI in particular has made these biomarkers useful for Arctic food web studies [43], which is termed IP_25_ [46]. IP_25_ is only produced in Arctic sea ice by four ice-associated diatom species [44,47]. HBI II, a similar compound with two double bonds, is co-synthesized with IP_25_ and serves as an additional suitable proxy for sea ice organic carbon [45]. In contrast, HBI III is produced by pelagic diatoms in open waters and ice-edge blooms [48], and serves as a pelagic counterpart. Based on the relative proportion of ice algae and phytoplankton utilized by primary consumers [49,50], the HBI biomarkers are incorporated into these lower trophic positions [42], and then subsequently into secondary consumer tissues through trophic transfer [43,51–53]. In marine mammals, the liver is metabolically active with high tissue turnover rates on the order of days to weeks [54]. While these short turnover rates were based on the stable isotope composition, studies of HBIs in marine mammal liver tissues relative to seasonal environmental conditions suggest that HBIs are metabolized on similar timescales [52,55]. Therefore, by analyzing HBI biomarkers in walrus livers, we can estimate carbon sources assimilated on short timescales that reflect recent sea ice conditions and associated primary production.

Understanding the ecological connections between the Pacific walrus and sea ice, and the associated potential threats owing to climate change, has been highlighted as an ongoing research imperative [25,56,57]. We hypothesize that sea ice organic carbon, derived primarily from ice algae production, is proportionally high for walruses (perhaps relative to other marine mammals in the region) owing to their foraging ecology and reliance on sea ice habitat. We would expect the walruses that closely follow the ice edge into the Chukchi Sea each spring would have proportionally greater amounts of sympagic HBIs in their diets overall. The objectives of this study were to (1) quantify the proportional contributions of ice algae and phytoplankton organic carbon sources to the diet of Pacific walruses with respect to the harvest location, time of year and associated sea ice conditions, (2) determine if males and females have differing proportions of sea ice organic carbon in their diet, (3) investigate differing proportions of sea ice organic carbon based on age class in the diet of walruses summering in the Chukchi Sea, and (4) evaluate sea ice organic carbon composition alongside the trophic position of Pacific walruses throughout the region to assess potential differences in the quality of Pacific walrus diets as a result of differing carbon sources available to their benthic prey.

## Materials and methods

### Study site

The geographic range of the Pacific walrus is confined to the shallow continental shelf of the Bering and Chukchi seas (Fig 1), with coastal haul-outs spanning many locations along the coasts of Russia and the United States (Alaska) [58]. Walrus samples in this study originated throughout their geographic range from several subsistence hunting communities. The villages of Gambell and Savoonga on St. Lawrence Island are positioned at the entrance of the Bering Strait, close to large wintering and breeding grounds in the NBS and along the passageway to the Chukchi Sea during the annual northern spring migration route [18,59]. Subsistence hunts occur here in the spring, typically around May of each year [60]. Walrus samples from the Chukchi Sea were provided from harvests that occurred near Peard Bay, Wainwright and Utqiaġvik while the animals were occupying their summer foraging grounds or in the autumn on their return south (Fig 1). The walruses harvested by hunters from the North Slope communities typically forage further offshore, on the shallow flanks of Hanna Shoal, but also near the coast when sea ice is low [4,11,12,22]. Point Lay is the location of large coastal haul-outs in the North American portion of the Chukchi Sea, particularly during low sea ice years [11,12]. Walrus liver tissues from Point Lay were collected as part of an earlier study, rather than subsistence hunting activities [61].

We used the Distributed Biological Observatory (DBO) sampling program to provide ecological context for the walrus tissue sampling. The DBO regions include five areas of high benthic biomass in the northern Bering and Chukchi sea, which in turn support higher trophic positions [9]. Several of these DBO regions are hotspots for walrus foraging activities owing to the high benthic biomass [4]. Our study focuses on DBO regions 1 and 4 (Fig 1). DBO 1 is located south of the St. Lawrence Island polynya, which is a key area for Pacific walrus wintering and a prominent breeding ground [18]. DBO 4 is located on the southeastern flanks of Hanna Shoal, which as described above is a shallow region on the northeast Chukchi shelf with substantial bivalve populations and the location of a prominent summer foraging ground for walruses [4,11,18].

### Sample collection

Walrus liver tissues were collected and donated from subsistence hunting activities in the NBS during the start of the spring migration (April/May) in 2012, 2014 and 2016 (Table 1). These harvests occurred from the villages of Gambell and Savoonga on St. Lawrence Island and from Diomede Village (Inalik) on Little Diomede Island (Fig 1). The NBS samples were stored frozen at -80°C at the University of Alaska’s Museum of the North (UAM). UAM provided 0.5 g tissue plugs, shipped on dry ice, which were freeze dried immediately upon arrival. The collection date, harvest location, sex, and age class of the walruses were documented in the UAM Mammal Collection database (Arctos; https://arctos.database.museum/home.cfm). Age class data were listed as unknown for all walruses sampled in 2012.

**Table 1:** Summary of Pacific walrus samples. The region and year of subsistence harvested Pacific walrus liver tissues with annual sample sizes (*n*) by sex. Annual sea ice persistence (days/year) associated with the year of collection and the sea ice organic carbon content (mean ± SE %) and stable nitrogen isotope composition *δ*^15^N (mean ± SD ‰) by sex. Mean sea ice organic carbon values were adjusted to account for a significant interaction between region and sex.

Region	year	sex	*n*	Sea ice persistence (days/year)	Sea ice organic carbon* (iPOC; mean ± SE%)	δ^15^N (mean ± SD‰)
**Northern Bering Sea** ^a^	2012	all	33	170	35 ± 3	13.3 ± 0.9
		males	10		17 ± 3	13.2 ± 1.2
		females	23		43 ± 4	13.3 ± 0.7
	2014	all	32	126	44 ± 3	13.0 ± 0.6
		males	24		40 ± 3	12.8 ± 0.4
		females	8		61 ± 5	13.6 ± 0.6
	2016	all	27	109	32 ± 3	13.5 ± 1.0
		males	14		30 ± 3	12.8 ± 0.5
		females	13		45 ± 3	14.3 ± 0.9
**Chukchi Sea** ^b^	2012	all	8	269	36 ± 6	14.5 ± 0.9
		males	3		35 ± 6	14.1 ± 0.8
		females	5		36 ± 8	15.0 ± 0.9
	2014	all	8	261	68 ± 6	15.2 ± 1.1
		males	5		71 ± 7	15.5 ± 1.2
		females	3		63 ± 8	14.7 ± 1.1

^a^Collected from Gambell, Savoonga and Little Diomede. Provided by the University of Alaska Museum of the North under permit number UAM 2018.020.Mamm.

^b^Collected from Peard Bay, Wainwright, Utqiaġvik and Point Lay. Provided by the North Slope Borough Department of Wildlife Management under US Fish and Wildlife Service permit number MA 80164B-0.

*Sea ice organic carbon mean values were adjusted to account for the interaction between region and sex.

Walruses harvested from the summer (June/July) foraging grounds in the Chukchi Sea were collected by subsistence hunters from Peard Bay, Wainwright, and Utqiaġvik in 2012 and 2014 (Fig 1). Additional liver samples from the Chukchi Sea were collected near Point Lay, which were necropsied as part of a large haul-out mortality investigation during October 2014. This sampling effort was jointly conducted by the community of Point Lay (Warren Harding and Leo Ferriera), the United States Fish and Wildlife Survey (Joel Garlich Miller and Jonathan Snyder), the North Slope Borough Department of Wildlife Management (NSB DWM; Raphaela Stimmelmayr and Isaac Levitt), and the Alaska Department of Fish and Game (Lori Quakenbush and Anna Bryan). These walruses were considered recently deceased (<1 week) based on external appearance and the state of internal organ preservation. The ambient temperature during the field investigation was cool (i.e. below and around freezing) with snow on the ground. These conditions resulted in carcasses that were naturally chilled, limiting organ decomposition. Therefore, degradation of HBIs and stable isotopes of nitrogen were concluded to be negligible and appropriate for this analysis [62,63]. The Chukchi Sea walrus samples were stored at -80°C by the NSB DWM (Table 1). Frozen walrus livers were shipped to the Chesapeake Biological Laboratory where 0.5–1 g of tissue was extracted using sterilized stainless steel scalpels followed by freeze drying over 24 hours. The collection date, location, sex, and age class of the walruses were provided from NSB DWM records.

### Sea ice analysis

Annual sea ice persistence data were calculated using sea ice concentration data from the National Snow and Ice Data Center (NSIDC, https://nsidc.org) Defense Meteorological Satellite Program (DMSP) Special Sensor Microwave Imager/Sounder (SSMIS), using a 15% threshold to determine the presence vs. absence of sea ice cover, following methods of Frey et al. [64]. Persistence data were parsed annually from September to September for each year to encompass the full seasonal sea ice advance and retreat cycle (e.g. 15 September 2011 through 14 September 2012). Mean annual sea ice persistence values were calculated for DBO 1 and DBO 4 for each year (Table 1). Sea ice persistence anomalies were calculated relative to the 1981–2010 average (i.e., the average of all years between 15 September 1980/14 September 1981 through 15 September 2009/14 September 2010) to assess broader spatio-temporal changes in sea ice by year. Anomalies were not reported for 2016 owing to the lack of associated walrus data from the Chukchi Sea.

Monthly averaged sea ice concentrations were retrieved at a 12.5-km resolution using the DMSP SSMIS passive microwave data (S1 Table). We calculated the mean monthly sea ice concentrations within the boundaries of DBO 1 or 4 associated with the time and location of the walrus harvest and their likely foraging grounds two weeks prior to determine a sea ice index (S1 Table). For example, a walrus harvested from St. Lawrence Island in early May was assumed to be foraging at DBO 1 in late April. DBO 1 was assigned as the foraging area for the NBS walruses harvested in the spring months and DBO 4 for the walruses harvested from the Chukchi Sea in the summer and fall months. However, we note that these assignments may not always accurately represent walruses from more southeastern foraging aggregations in the St. Lawrence Island/Diomede harvest or walruses recently migrating north in the early summer (June) harvests from Utqiaġvik/Wainwright [11]. To account for the potential influence of variable distributions, and to meet the assumptions of independent data, we calculated the mean of the sea ice organic carbon values associated with sea ice concentrations or sea ice persistence for additional analyses.

### Biomarker extraction and analysis

After freeze drying, HBIs were extracted following established methods [45,65]. Samples were saponified in a methanolic KOH solution and heated at 70°C for one hour. Hexane (4 mL) was added to the saponified solution, vortexed, and centrifuged for three minutes at 2500 RPM, three times. The supernatant with the non-saponifiable lipids (NSLs) was transferred to clean glass vials and dried under a gentle N_2_ stream. The initial extracts were re-suspended in hexane and fractionated using open column silica gel chromatography. The non-polar lipids containing the HBIs were eluted while the polar compounds were retained on the column. The eluted compounds were dried under N_2_. 50 μL of hexane was added twice to the dried purified extract and transferred to amber chromatography vials.

The extracts were analyzed using an Agilent 7890A gas chromatograph (GC) coupled with a 5975 series mass selective detector (MSD) using an Agilent HP-5ms column (30 m x 0.25 mm x 0.25 μm), following established methods [65]. The oven temperature was programmed to ramp up from 40°C to 300°C at 10°C/minute with a 10-minute isothermal period at 300°C. HBIs were identified using selective ion monitoring (SIM) techniques. The SIM chromatograms were used to quantify the HBI abundances by peak integration with ChemStation software (Agilent Technologies, California, USA). A purified standard of known IP_25_ concentration was used to confirm the mass spectra, retention time and retention index (RI). The HBIs were identified by their mass ions and RI including IP_25_ (*m/z* 350.3), HBI II (*m/z* 348.3) and HBI III (*m/z* 346.3). A procedural blank was run every 9^th^ sample.

We used the H-Print index (Eq 1) to provide an estimate of the relative organic carbon contributions of phytoplankton to sea ice algae [45], using the abundances of IP_25_, HBI II and HBI III:

$$H‐\mathrm{Print}\%=\frac{\mathrm{HBI}\;\mathrm{III}}{IP_{25}+HB\;III+HBI\;III}x\;100$$ (1)

Lower values are indicative of proportionally greater sympagic organic carbon and higher values are indicative of proportionally greater pelagic organic carbon. Sea ice organic carbon (iPOC), as a proportion of marine-origin carbon within samples, was estimated using a prior H-Print calibration determined from feeding experiments with known algal species (R^2^ = 0.97, *p* <0.01, df = 23 [66]).


iPOC%=101.8–1.02xH‐Print (2)

In contrast to the H-Print index, higher iPOC values reflect greater proportions of organic carbon derived from ice algae. We define iPOC values exceeding 50% as indication of elevated sea ice organic carbon in the animal’s diet.

### Nitrogen stable isotope analysis

Freeze-dried samples were homogenized with a mortar and pestle. 0.4–0.6 mg of tissue was weighed and introduced into tin capsules. The nitrogen stable isotope ratios were measured at the stable isotope facilities at the Chesapeake Biological Laboratory using a Costech elemental analyzer coupled to a ThermoFisher Delta V Isotope Ratio Mass Spectrometer operated in a continuous flow mode. Nitrogen isotope ratios are expressed relative to atmospheric nitrogen (N_2_) using the following equation:

$$\delta X=[(\frac{R_{sample}}{R_{standard}})-1]x\;1000$$ (3)

where R is the corresponding ratio of ^15^N/^14^N. Accuracy of the data was assured by simultaneous combustion and analysis of the samples with an internal standard of acetanilide, run every 9 samples, which has a well-characterized isotopic composition. Precision was also assessed by analysis of this internal standard. Samples were analyzed on two separate dates and the precision of the acetanilide standard run simultaneously was ±0.21‰ on the first date (n = 5) and ±0.08‰ on the second date (n = 6). The mean *δ*^15^N value determined for acetanilide for both dates were within 0.12‰ of each other.

### Statistical analysis

All analyses were conducted using R version 3.6.1 (www.r-project.org). Data were normally distributed with equal variances. A significance level of α = 0.05 was used for all tests. We explored the regional influence of sympagic and pelagic HBIs graphically through non-metric multidimensional scaling (NMDS) ordination plots using the R package ‘vegan’ [67] to look for distinctions in carbon sources associated with the walrus harvest location and associated sea ice conditions. Distances were based on Bray-Curtis dissimilarities of the three measured HBIs (IP_25_, HBI II and HBI III). We assigned sea ice organic carbon (iPOC), H-Print, and sea ice persistence as the environmental vectors (noting that iPOC and H-Print are calculated from the HBIs and the direction of the vectors suggest stronger associations with either ice algae or phytoplankton, respectively). We conducted a two-way ANOVA test with sex and region as factors. Both factors and the interaction of the two were significant (*p* < 0.01). To account for this interaction, the means were adjusted by grouping sexes among each year and comparing iPOC values by region using the R package ‘lsmeans’, which were reported with standard error (SE). We then carried out planned comparisons using the Bonferroni correction for multiple comparisons to detect differences in mean iPOC values between regions, sex and years. Given the limited age class data associated with the walruses harvested in the NBS, the effect of age class on sea ice organic carbon estimates could only be adequately examined for the Chukchi Sea. The age class analysis was therefore conducted using a Welch two sample t-test to examine the difference between adults and calves.

Trophic positions were estimated with a Bayesian model approach from the nitrogen stable isotope values using the R package ‘tRophicPosition’, which uses Markov Chain Monte Carlo simulations [68]. This Bayesian approach uses relevant statistical distributions around the consumer and baseline observations to allow these values to be treated as random variables. We used the trophic enrichment factor (TEF) estimate for each trophic position that is based on Post’s widely utilized meta-analysis (3.4 ± 0.98 [69]). While there are also species- and tissue-specific TEFs to consider [70–72], the modelling approach incorporates uncertainty in the TEFs around our estimation of relative trophic position (hereafter referred to as ‘trophic position’ or ‘TP’). We selected one baseline for the model, which links the *δ*^15^N enrichment per trophic level with the trophic positions of the baseline (in this case, a primary consumer) [68]. For the baseline, we utilized previously published *δ*^15^N values for *Macoma calcarea* collected from Hanna Shoal in 2012 [73] and from DBO 1 and DBO 4 in 2015 [74] to serve as our baseline consumer in the model (S2 Table). These baseline values were temporally and spatially consistent with the Pacific walrus harvests in this study. The *M*. *calcarea* values were comparable between the two studies (*δ*^15^N = 10.1 ± 0.9, *n* = 36 [73]; *δ*^15^N_DBO 1_ = 9.6 ± 0.5 and *δ*^15^N_DBO 4_ 9.3 ± 0.8 [74]) and allowed us to determine a reasonable estimate of the variability. The median trophic position estimates from the model were reported along with the 95% confidence levels. Pairwise comparisons of posterior trophic positions estimates were assessed to determine significant differences between regions and sexes. This model allows for the input of *δ*^13^C values for running mixing models (particularly when using two baselines) to generate carbon versus nitrogen plots to examine basal food sources and consumers spatially. The *δ*^13^C values were excluded from our study owing to the influence of lipids in our samples and inability to correct for their presence [75]. However, for the purpose of exploring associations sea ice organic carbon contributions, iPOC values were substituted for *δ*^13^C in the model, which is not used in the calculation of the TP estimates with one baseline and does not impact these values (S1 Table). To do this, we used mean iPOC estimates for *M*. *calcarea* from a prior investigation of HBIs in benthic invertebrates from DBO 1 (6.2 ± 5.4%, n = 4) and DBO 4 (35.6 ± 6.8, n = 7) [42]. Based on the results of that study, we assumed that there would be comparable iPOC values from year to year based on similar annual distributions of HBIs in the surface sediments and similar HBI values between *M*. *calcarea* and surface sediments. We also employed a one-way ANOVA test to explore the relationship between sex, region and nitrogen stable isotope values.

## Results

### Sea ice

**Fig 2:**
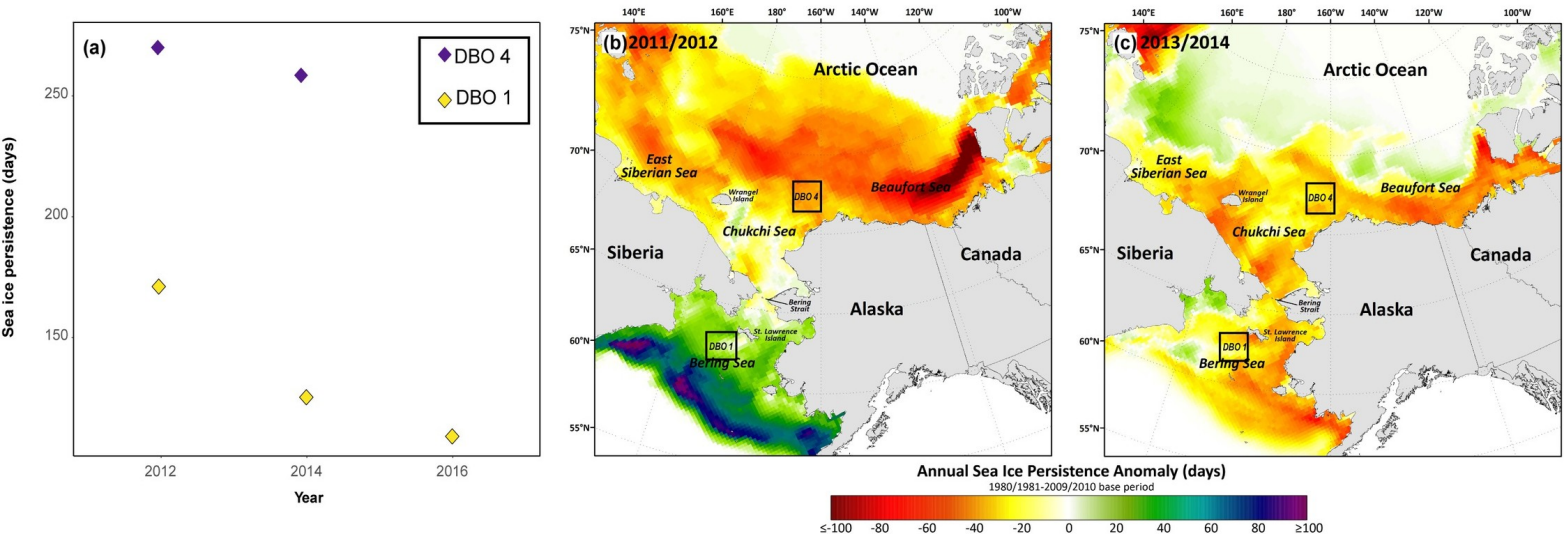
Sea ice persistence in the Pacific Arctic. **a)** Sea ice persistence (days/year) by year for the northern Bering Sea and Chukchi Sea, **b)** Sea ice persistence anomalies for 2012 (15 September 2011–14 September 2012), **c)** Sea ice persistence anomalies for 2014 (15 September 2013–14 September 2014). Made with Natural Earth coastline data. Sea ice persistence anomalies initiate in September for the preceding calendar year to include sea ice formation and retreat in one sea ice season and were compared to a 1980/1981-2009/2010 base period.

Sea ice persistence progressively declined in the NBS throughout the study period from 170 days/year in 2012, to 126 days/year in 2014, and 109 days/year in 2016; an overall decline of 61 days (36%) (Fig 2A). In contrast, sea ice persistence was similar between 2012 (269 days/year) and 2014 (261 days/year) in the Chukchi Sea (Fig 2A). Sea ice persistence anomalies were calculated to look at broad regional trends in 2012 and 2014, revealing a divergent pattern throughout the Pacific Arctic in 2012 (Fig 2B). Sea ice persistence at DBO 1 (NBS) was approximately 21 days above the 1981–2010 average, while sea ice persistence was approximately 34 days below average at DBO 4 (Chukchi Sea; Fig 2B). In contrast, sea ice persistence was uniformly below average at DBO 1 (-21 days) and DBO 4 (-31 days) in 2014 (Fig 2C).

**Fig 3:**
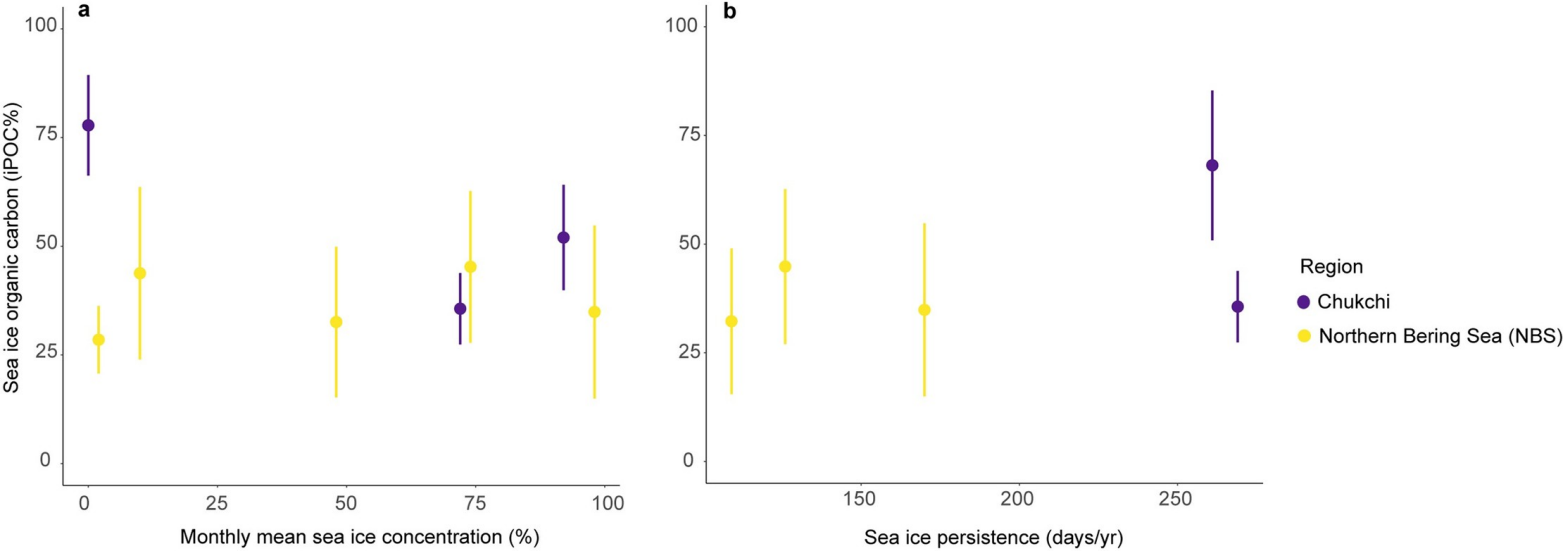
Sea ice indexes and sea ice organic carbon. **(a)** Monthly mean sea ice concentration (%) and mean sea ice organic carbon. Individual points represent the mean sea ice organic carbon with standard deviation. **(b)** Sea ice persistence (days/year) and sea ice organic carbon.

Assigned monthly mean sea ice concentration values ranged from 0–98% overall (S2 Table). In 2012, walruses harvested in the NBS in May were associated with a 98% (April) monthly mean sea ice concentration. Walruses harvested in July from the Chukchi Sea were associated with a 72% (June) monthly mean sea ice concentration. For 2014 in the Chukchi Sea, mean sea ice concentrations associated with the walrus harvest dates were 0% (September) for walruses sampled in October and 92% (June) for walruses harvested in July. For the NBS in 2014, walruses harvested in early May were associated with a 74% monthly mean sea ice concentration in April and dropped to 10% in May for walruses harvested in late May. During April of 2016, the month prior to walrus harvests in the NBS, the mean sea ice concentration was 48%. There were no significant relationships between either satellite-derived sea ice parameter and sea ice organic carbon based on linear regressions (Fig 3). As stated previously, linear regressions were based on the mean iPOC values associated with specific sea ice concentration or sea ice persistence values, rather than the individual data points.

### Sea ice organic carbon (iPOC%) by region, sex and age class

**Fig 4:**
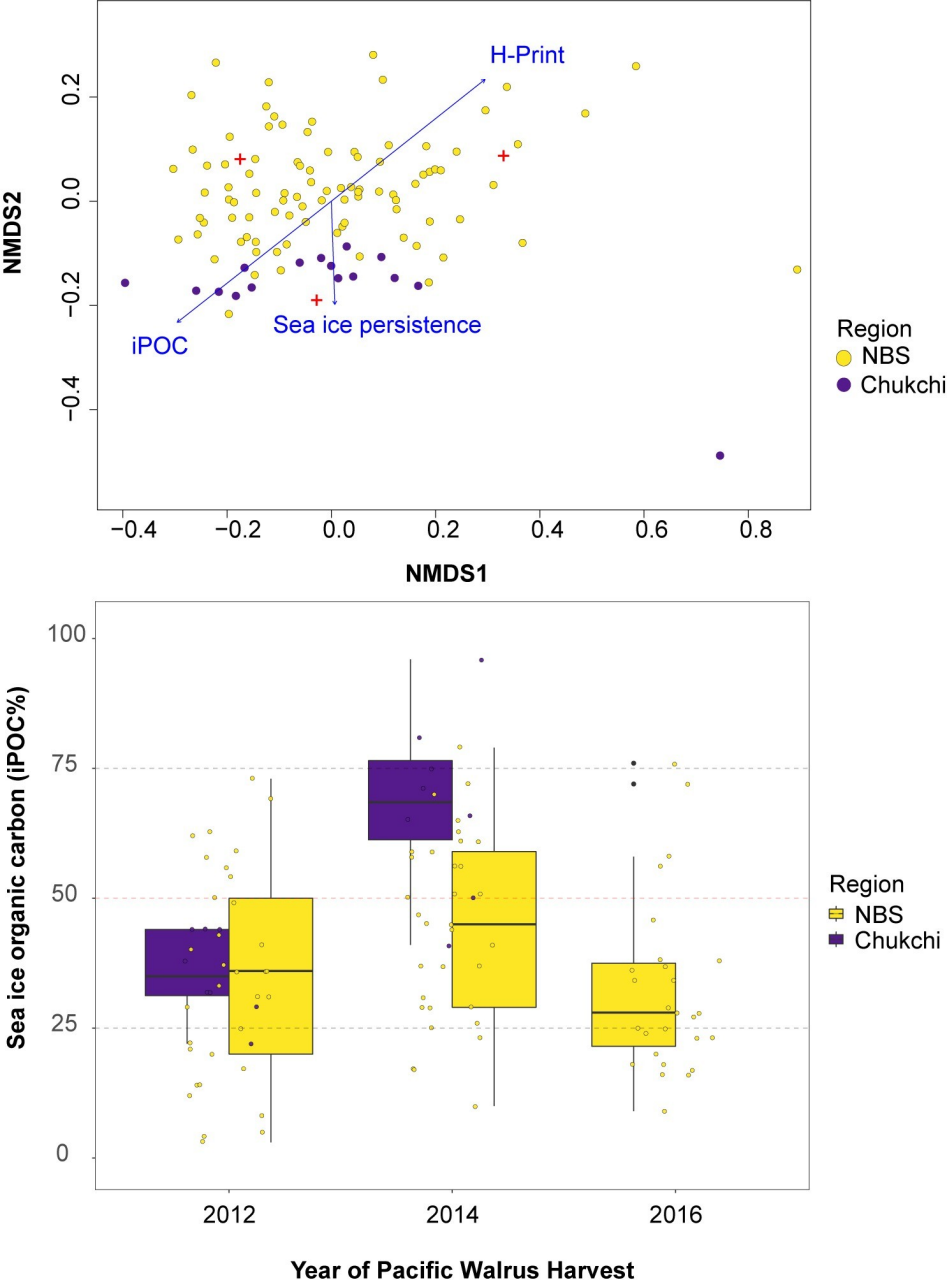
Regional analysis of sea ice organic carbon. **a)** Ordination plots based on the individual highly branched isoprenoids (HBIs), including IP_25_, HBI II and HBI III, used to calculate sea ice organic carbon (iPOC) content. Data points are colored by region and the considered environmental vectors as blue arrows. **b)** Boxplots for iPOC (%) by year and region. Boxes depict the interquartile range from the first to third quartiles, including the median (horizontal line), minimum/maximum (vertical lines) and outliers (individual points).

The NMDS ordination plot indicated there were distinct groupings in sea ice organic carbon content between walruses in the NBS and Chukchi Seas (Fig 4A). The environmental vectors (iPOC, H-Print and sea ice persistence) indicate a stronger association with ice algae contributions (i.e. iPOC) and sea ice persistence on the Chukchi Sea walruses while there were greater associations with phytoplankton contributions (i.e. H-Print) on the NBS walruses. Two-way ANOVA tests indicated that region was a significant factor (DF = 1, F = 8.32, *p*<0.01), as well as sex (DF = 1, F = 10.51, *p*<0.01). The adjusted iPOC values (mean± SE) in livers were highest overall in the Chukchi Sea with mean female values of 46 ± 6% and male values 58 ± 6%. In the NBS, mean iPOC values for females were 45 ± 3% and 30 ± 3% for males (Fig 4B, Table 1).

**Fig 5:**
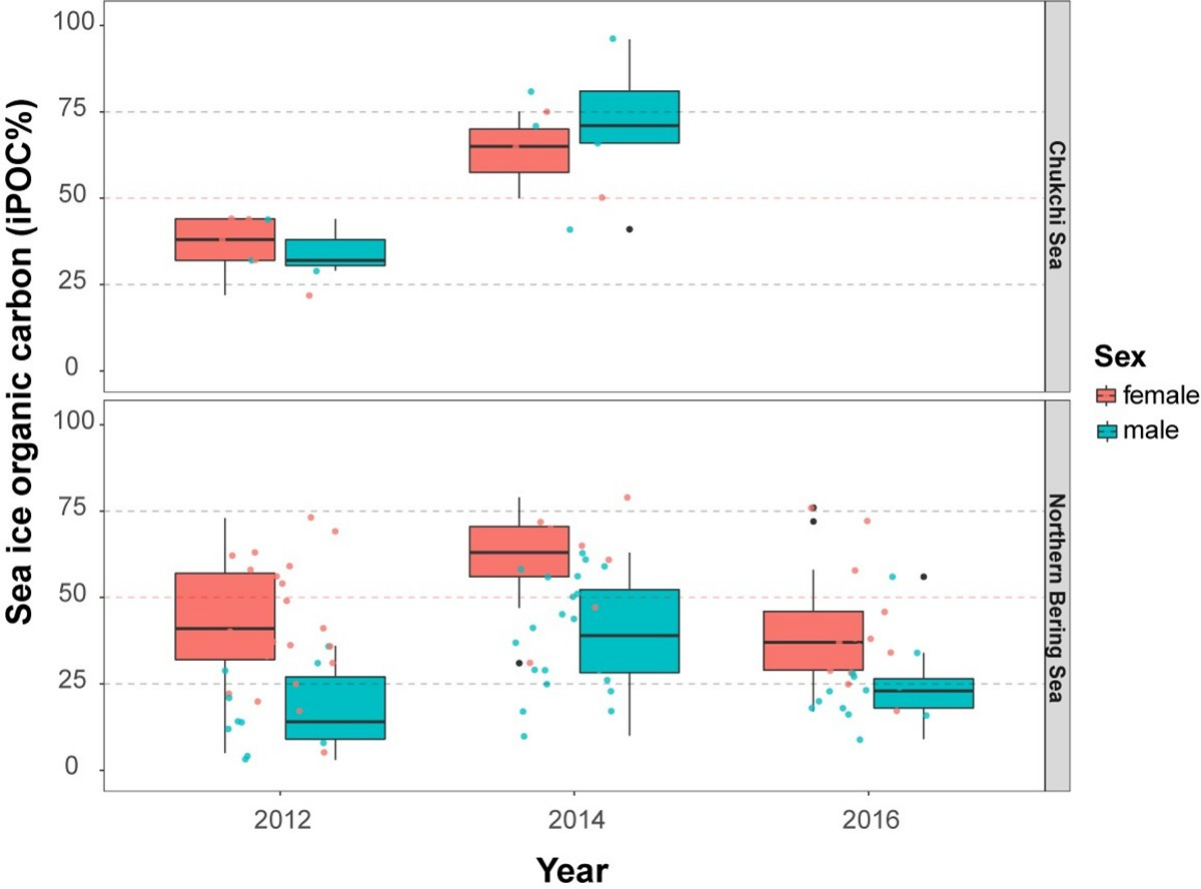
Sea ice organic carbon by region and sex for 2012, 2014 and 2016 (NBS only). The boxplot indicates the interquartile range from the first to third quartiles, with the median shown as the horizontal line within each box for females and males for each region. All individual data points are shown. The red-dashed line indicates 50% sea ice organic carbon utilization, with values above this level suggesting elevated sea ice organic carbon.

Pairwise planned comparisons confirmed sea ice organic carbon values were significantly higher in NBS females when compared to NBS males for all 3 years of this study (*p* < 0.001; Fig 5). In contrast, sea ice organic carbon values were similar (*p*>0.05) between males and females in the Chukchi Sea in both 2012 [35 ± 6% (males) and 36 ± 8% (females); Table 1] and 2014 [71 ± 7% (males) and 63± 9% (females); Table 1]. However, mean iPOC values in walruses were significantly different between 2012 (36 ± 6%) and 2014 (68 ± 6; *p* < 0.001) (Table 1) in the Chukchi Sea, while values were comparable between 2012, 2014 and 2016 in the NBS. In 2014, the mean iPOC values were significantly different between the Chukchi Sea (68 ± 6%) and NBS (44 ± 3%), but were comparable in 2012 (36 ± 6% and 35 ± 3%, respectively) (Table 1). When factoring in both sex and region, the differences were only observed among the males and not the females. In 2012, Chukchi Sea males (35 ± 6%) had higher values than NBS males (17 ± 3%; *p* < 0.05; Table 1). In 2014, Chukchi Sea males (71 ± 7%) were again significantly greater than NBS males (40 ± 3%, *p* < 0.001).

**Fig 6:**
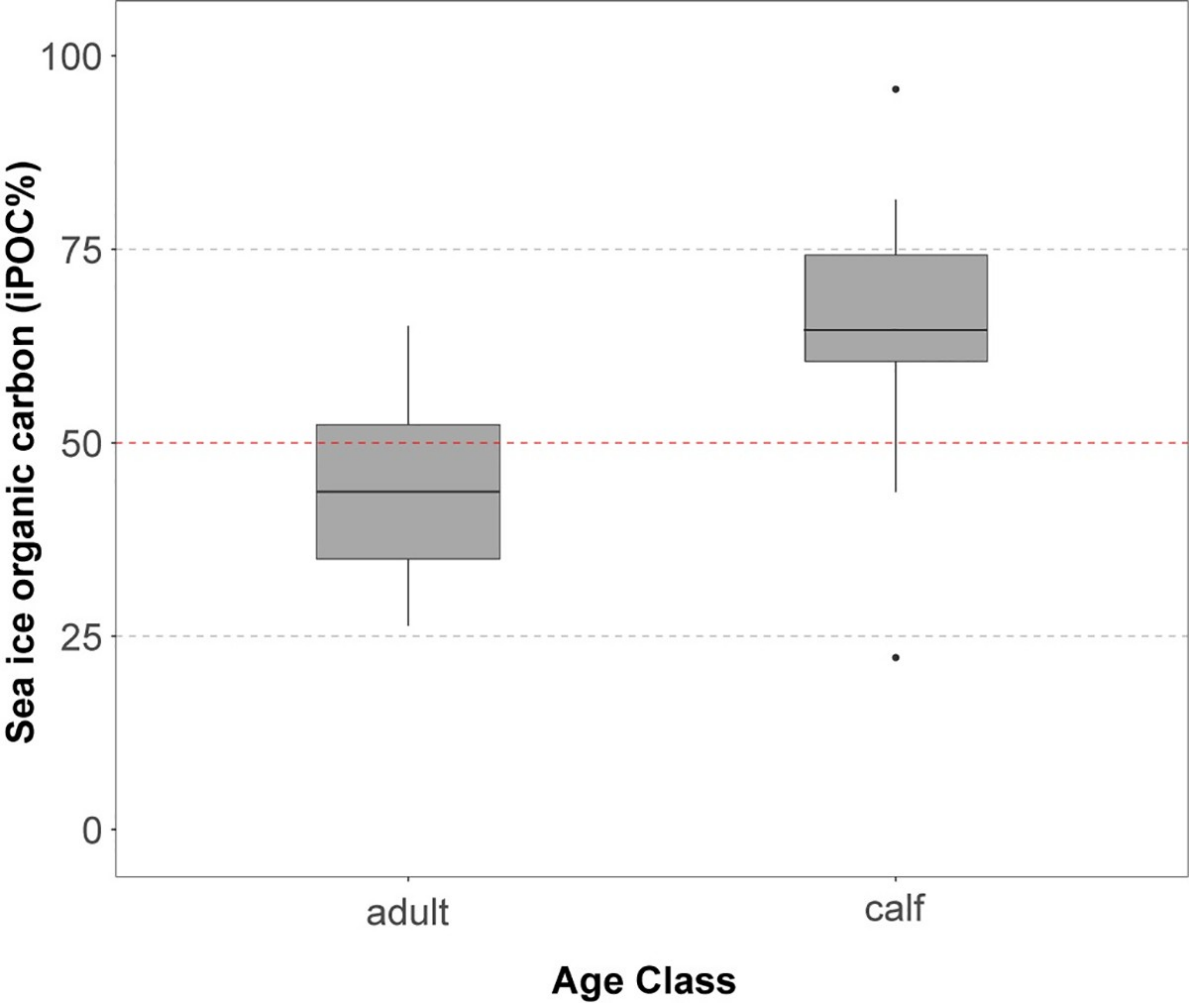
Sea ice organic carbon (iPOC%) by age class in the Chukchi Sea. Box plots represent the interquartile range of values for adults (*n* = 9) and calves (*n* = 7) in the Chukchi Sea. The red-dashed line indicates the 50% sea ice organic carbon threshold, with values above this level suggesting elevated sea ice organic carbon contributions to the Pacific walrus diet.

A Welch two-sample t-test indicated the difference in iPOC values between calves and adults in the Chukchi Sea was significant (t_8_ = -2.8, *p*<0.05). Sea ice organic carbon values in calves was 65% ± 25% (mean ± SD, *n* = 7) versus 42% ± 11% (*n* = 9) in adults (Fig 6). Six of the seven walrus calves had liver iPOC values that exceeded 50%, the threshold that suggests proportionally greater contributions of sea ice algae at the base of their diet.

### Stable nitrogen isotope composition and trophic positions

Overall, *δ*^15^N values ranged from 11.9‰ to 16.9‰. In the NBS, *δ*^15^N values for females were 13.3 ± 0.7‰ in 2012, 13.6 ± 0.6‰ in 2014 and 14.3 ± 0.9‰ in 2016 (mean ± SD, Table 1). For males, *δ*^15^N values were 13.2 ± 1.2‰ in 2012, 12.8 ± 0.4‰ in 2014 and 12.8 ± 0.5‰ in 2016 (Table 1). In the Chukchi Sea, mean *δ*^15^N values for females were 15.0 ± 0.9‰ in 2012 and 14.7 ± 1.1‰ in 2014 (Table 1). For males, *δ*^15^N values were 14.1 ± 0.8‰ in 2012 and 15.5 ± 1.2‰ in 2014 (Table 1). Sex and region were both significant (*p* < 0.001) factors for *δ*^15^N values as determined by a two-way ANOVA test. The interaction of the two factors was not significant (*p* > 0.05). The pairwise comparisons show each group was significantly different, with the exception of Chukchi males versus females.

The TP estimates were similar between regions and sexes. Overall, TPs were minimally but statistically higher in the Chukchi Sea at 3.5 compared to 3.2 in the NBS (Table 2). In the NBS, TPs were similar for females and males (3.2 and 3.1, respectively; Table 2). Median TP estimates were the same for males and females in the Chukchi Sea (3.5; Table 2). Posterior pairwise comparisons indicated males in the NBS and Chukchi Sea were significantly different (*p*<0.05).

**Table 2:** Trophic position (TP) estimates for Pacific walruses by region and sex. TP estimates are shown as the median with 95% confidence levels (CL). Regions included the northern Bering Sea (NBS) and Chukchi Sea. The values shown in bold and italicized were significantly different (*p*<0.05).

	Northern Bering Sea	Chukchi Sea
**All**		
Lower 95% CL	3.0	3.3
Median	***3*.*2***	***3*.*5***
Upper 95% CL	3.3	3.7
**Females**		
Lower 95% CL	3.1	3.1
50%	3.3	3.5
Upper 95% CL	3.5	3.9
**Males**		
Lower 95% CL	2.9	3.1
Median	***3*.*1***	***3*.*5***
Upper 95% CL	3.3	3.9

## Discussion

### Higher sea ice organic carbon in the summer diet of Pacific walrus in the Chukchi Sea

The higher contribution of sea ice organic carbon in the Pacific walrus tissues from the Chukchi Sea compared to the NBS (Figs 4 and 5) are consistent with sea ice persistence and HBI distributions previously determined from surface sediments [76]. Those HBI measurements were assumed to reflect differences in ice algae production and deposition throughout the Pacific Arctic region [76], largely driven by sea ice persistence, where sea ice is typically present 2–4 months longer in the Chukchi Sea [64]. A recent study of particle fluxes in the northeast Chukchi Sea identified year-round export of diatoms (including ice-associated diatoms) on the shallow shelf, which suggested ice algae could be a sustaining resource for benthic communities in the less productive months [77]. Additionally, subsurface chlorophyll measurements alongside disassociated ice algae also suggest that ice algae are likely contributing to prolonged periods of productivity on the Chukchi shelf [78]. These data imply that sympagic HBI levels in the northeast Chukchi Sea would be higher than those found in the NBS, and therefore the sea ice organic carbon would be higher in the prey items utilized by northerly walrus populations in the summer and autumn. The sea ice organic carbon values in Pacific walrus livers presented here likely reflect the broader HBI distributions observed in the environment and the location of primary foraging activities rather than differences in resource selection.

As stated previously, Pacific walruses are central place foragers that primarily consume bivalves [18], which often dominate benthic macrofaunal biomass across the region [4]. The sea ice organic carbon values in benthic invertebrates across the Pacific Arctic, specifically in Tellinid bivalves [42], are consistent with this hypothesis. These bivalves are ubiquitous throughout the entire region and generally matched the HBI distribution patterns in the surrounding surface sediments, with elevated sea ice organic carbon relative to the surface sediments in a few locations [42]. Tellinid biomass has proven to be a significant predictor of Pacific walrus resource selection models in both the northern Bering [26] and Chukchi [32] seas and therefore *M*. *calcarea* is a useful representative species for tracking sea ice organic carbon contributions to the Pacific walrus diet. While iPOC data in Tellinid clams are currently unavailable to correspond with the timing of the walrus harvests and samples used in this study, mean iPOC values from both regions in 2018 suggest a significant difference in their sea ice organic carbon content (~6% in the Bering Sea and ~40% in the Chukchi Sea) [42]. Given the similarities between males and females in the Chukchi Sea walrus samples, the prey HBI data suggest these walruses likely have similar foraging behaviors. The study assessing sea ice biomarkers in benthic fauna also revealed elevated sea ice organic carbon use by another common walrus prey item, sipunculid worms [42], which are also more prevalent in the Chukchi Sea than in the NBS [79]. If the availability of certain preferred prey items in the Chukchi Sea declines in the future because of this apparent dependence on ice algae, there will likely be consequences for Pacific walruses [79]. However, it remains to be seen how benthic organisms respond to shifting carbon sources.

We found no significant relationship between the two sea ice parameters tested (monthly mean sea ice concentration and sea ice persistence) with iPOC values in Pacific walrus tissue. However, this may be unsurprising owing to the fact that this species migrates across a gradient of sub-Arctic to Arctic habitat with distinct sea ice conditions, while also coupling the benthic and pelagic oceanic realms along the way. These large-scale movements likely result in both horizontal and vertical habitat integration. This integration across the entire Pacific Arctic region over the course of a few months during the ice melt season is likely why our sea ice index did not robustly match iPOC values in the walrus liver tissues. The elevated sea ice organic carbon values measured in the Chukchi Sea walruses collected during open water periods (October) exemplify the nuances of analyzing associations with satellite-derived sea ice data. These walruses had a longer period of time to forage in the Chukchi Sea, consuming more of the prey that had previously assimilated sea ice organic carbon than those harvested earlier in the summer as the ice edge retreated. We conclude that direct associations between these biomarkers and sea ice data (e.g. concentration, persistence, or break-up date) in migratory consumers in this region may not always be possible and require further interpretation and perhaps paired with additional trophic markers (e.g. fatty acids, compound-specific stable isotopes).

Despite the lack of association between the sea ice parameters and sea ice organic carbon, there were interesting patterns within a broader context when considering the sea ice persistence anomalies from 2012 and 2014. For 2012, the NBS was a cold year in which sea ice persistence was well above average (Fig 2B). However, there was also a geographically divergent pattern in 2012, as sea ice in the Chukchi Sea was a record low year [64] (Fig 2B). This geographically divergent sea ice pattern would potentially explain higher than average sea ice algae trophic markers in the Bering Sea in 2012, potentially reflected in comparable values for the two regions (Fig 5B). However, in 2014, sea ice persistence anomalies indicate similarly negative values throughout the region (Fig 2B). Therefore, the default HBI distribution pattern observed for the region (increasing sympagic HBIs from south to north [76]) was likely reflected in consumer tissues. This broad assessment supports the use of HBIs as fingerprints of where large marine predators were foraging, similar to the way that carbon isotope distributions have been used to create isoscapes that map differences in the intensity of primary production throughout a given area [80]. Additionally, tracking sea ice organic carbon in these organisms over time may still be useful in monitoring responses to declining sea ice. HBI measurements of other marine mammals and seabirds in this region with differing foraging ecologies will be potentially useful in further assessing changes in food webs as seasonal sea ice declines.

The effects of declining sea ice on Pacific walruses may already be evident in the Bering Sea. Observations from the southwestern Bering Sea indicate that occupation of land haul-outs by males in this region may be shifting to northward locations in the Chukchi Sea on the Chukotka Peninsula [81]. There have also been more mixed-sex herds observed in the Chukchi Sea [25]. Coincident shifts with their prey base are also occurring as indicated by a northward shift in bivalve populations at DBO 1 [28], which is near a prominent walrus breeding ground and a prey base for these walruses [26]. In addition to declining sea ice, the northward shift in bivalve biomass will also likely influence the Pacific walrus haul-out locations to move north. Negative associations with sea ice declines have also been reported in body condition observations for females and juveniles of other pinniped species in this region [82]. These observations together suggest a broader ecosystem shift is underway in response to warming waters, reduced sea ice coverage and timing of ice retreat, and changes in the quality, quantity and timing of primary production [5,29].

In a recent similar study using HBIs in Atlantic walruses (*O*. *r*. *rosmarus*) in the Canadian Arctic, coupled with measurements of *δ*^13^C_phe_ (phenylalanine–a source amino acid), suggested nearly exclusive sea ice-derived carbon as the base of the walrus diet at high-latitudes (89–98% iPOC) [83]. These values were significantly higher than Atlantic walruses examined from lower latitude locations, suggesting differences in carbon energy sources based on location and changing ice algal phenology at lower latitudes. This study also observed a 75% decrease in sea ice organic carbon over two decades in the lower latitude walruses that was strongly associated with declining sea ice, which was not observed with the similar decline in sea ice at high latitudes. While these two subspecies differ in their migratory behavior (Pacific walruses follow the ice-edge and Atlantic walruses do not), their findings regarding regional distinctions in sea ice-derived carbon are similar to what we observed in this study. However, the iPOC values in the Atlantic walruses were notably higher than most of the values observed in the Pacific walruses of this study, which is consistent with the higher persistence of sea ice in the Canadian Arctic. The differences in migratory behavior are also probably why associations between the Atlantic walrus iPOC values and satellite-based sea ice observations were stronger. Additionally, they concluded that shifts in ice algal phenology are likely leading to a decoupling of sympagic-benthic systems and impacting benthic consumers, which is also a likely explanation for the changes in dominant benthic organisms (biomass and abundance) and northward migrations underway in the northern Bering Sea ecosystem [28,29].

### Higher sea ice organic carbon in female versus male Pacific walrus diets in the northern Bering Sea

The contribution of sea ice derived organic carbon was higher in females than in males in the NBS, whereas there was no difference between sexes in the Chukchi Sea (Fig 4). This can be explained by several factors. First, the foraging behavior of walruses may be different between males and females in winter and spring when walruses are in the NBS, but similar in summer when they are in the Chukchi Sea. Early studies of walrus stomachs suggested that females and juveniles tended towards smaller bivalves compared to that of males [18]. Reported sea ice organic carbon values among various feeding strategies of benthic invertebrates throughout the Pacific walrus range suggested elevated ice algae consumption by subsurface deposit feeders and elevated phytoplankton consumption by suspension feeders and predator/scavengers [42]. If female walruses spend greater efforts foraging on infaunal organisms deep in the sediments rather than on motile epifauna, including predatory gastropods or crustaceans, this may be one explanation for differences in their sea ice organic carbon levels compared to males. Differences in male and female prey selection have been previously reported. For example, male Pacific walruses may opportunistically forage on higher trophic level prey, including seals and seabirds, if there is a lack of preferred prey owing to availability or requirements to forage in nearshore environments [18,84]. Indications of seals and other higher trophic level prey in walrus diets have been suggested through stable isotopes and is supported to some extent with observational data [85]. However, this behavior, and whether it differs between males and females, remains inconclusive but is believed to be atypical [18,84]. The similar trophic position estimates between males and females observed in this study do not indicate this distinct behavior occurs by one sex (Table 2). Owing to the primarily female migration during the summer and autumn seasons, general differences in diet by sex can be anticipated based on the prey availability in different foraging areas, particularly with males foraging from coastal haul-outs and females foraging from offshore sea ice. It had previously been concluded that males and females generally eat the same prey while occupying the same area [13,86,87].

Second, to support lactation and pregnancy, females may show selective foraging on more lipid-rich food sources and increase their foraging effort to support the energetic demands during lactation [36]. Females have been shown to double clam consumption during this time [86]. A higher consumption rate might result in a greater contribution of sea ice derived carbon in the diet of females compared to males. Resource partitioning may also be linked to breeding, which was implicated as the reason for differences in fatty acids measured in Pacific walrus blubber [88]. During the winter breeding season in the NBS, male walruses reduce foraging efforts to focus on mating [89]. The breeding season for the Pacific walrus occurs approximately from January through March [18]. The walrus livers from the NBS included in this study were collected in April and May (Table 1). Based on the assumed turnover rate of HBIs in liver, it is possible that the lower sea ice biomarker values are a reflection of this behavior.

The higher contribution of sea ice carbon in female walrus during the breeding period strongly suggest an elevated reliance of females to sea ice during this period compared to males. The results from Oxtoby et al. [88] suggested an incorporation of lipid-rich resources from the previous season, reflecting turnover rates of several months for blubber. By contrast, the use of liver in this study and a more rapid turnover rate further constrains this possible resource partitioning to the time period close to the breeding season and subsistence hunt. By reducing the time period of metabolic activity, we conclude the dependence on sea ice organic carbon is not derived from a prior year. With ongoing sea ice decline, females are likely to be at greater risk of disturbance than males [22,36,56,90]. The loss of sea ice could impact the females’ ability to successfully reproduce and provide adequate nutrition for their offspring [12,36,86]. Stress and reproductive biomarkers analyzed from recent and archaeological Pacific walrus bones suggested potential resilience to declining sea ice [91], but ongoing monitoring of this species is necessary to track their response to unprecedented change.

### Similar trophic position and *δ*^15^N for Pacific walruses despite their seasonal distributions

*δ*^15^N values of bulk tissue in predators is a useful tool to estimate trophic position but is influenced by changes in *δ*^15^N values at the base of the food web, or baseline. Here, we estimated the trophic position of Pacific walruses taking in account regional values of the *δ*^15^N at the baseline (*i*.*e*. primary consumers), allowing comparison between the two regions. Trophic position estimates were similar between regions and sexes, with median values ranging from 3.1 to 3.5, which were in alignment with expected values (~3.3; [83]). The model output indicated the difference was significant between the NBS (3.2) and Chukchi Sea (3.5) overall, but this difference is not particularly meaningful in terms of food web structure (Table 2). Based on previous studies of stomach contents, these TP estimates are in agreement that the summer and autumn diets of Pacific walruses in the Chukchi Sea are not substantially different than those in the NBS in the spring and winter [13]. The distribution and availability of bivalves, their preferred prey item, throughout the Pacific Arctic region supports this conclusion [4]. The trophic position estimates for Pacific walruses were also similar to those reported for Atlantic walruses (2.8–3.5), which also primarily feed on bivalves [83]. Based on comparable trophic positions between the Bering and Chukchi Sea, the observed elevation in sea ice organic carbon values in the Pacific walrus liver tissues from the Chukchi Sea reveals an increasing contribution, and perhaps significance, of sea ice algal production at the base of the food web on a latitudinal gradient. Our estimates do not provide evidence of higher trophic level prey consumption (i.e. seals), which has been reported to occur opportunistically with this species [84].

The *δ*^15^N values of bulk tissue of Pacific walrus liver tissues from this study agreed with previous liver tissue stable isotope measurements made from Pacific walruses harvested from St. Lawrence Island (13.0 ± 1.2 ‰) [84]. We found higher *δ*^15^N values in walruses from Chukchi Sea compared to the Bering Sea, that could be driven by differences at the base of the food web, as we found no regional differences in TP when taking in account the *δ*^15^N baseline. The higher *δ*^15^N values at the base of the Chukchi sea food web can be a result of several processes. The food web in the Chukchi Sea becomes more complex than the NBS as a result of the Pacific water inflow system, where advective processes deliver substantial carbon sources that are in part degraded and deposited as currents move across the shelf [92]. With these inputs, the detrital food web becomes more influential in the northern Chukchi, which could lead to higher *δ*^15^N values in the baseline. The hydrography of the Chukchi Sea around Hanna Shoal has been shown to influence the benthic food web, with increased benthic-pelagic coupling under nutrient rich Anadyr water [37]. As a result, benthic denitrification can impart an enriched *δ*^15^N signal in the water column, a process called “sedimentary isotope effect” [93,94]. This effect has been previously observed in the benthic food web in the Chukchi Sea [95]. The advection of degraded material inputs is also evident in the observed gradation of denitrification in the water column moving from south to north in this region [96].

## Conclusions

Our study sought to reveal linkages between sea ice algae and Pacific walrus diets, as shifts in primary production driven by climate change will have cascading effects on the food web. HBI biomarkers revealed distinct differences in sea ice organic carbon between walruses sampled from 2012, 2014 and 2016 that varied between sex and region. Sea ice organic carbon was higher in walruses that migrated into the Chukchi Sea and were collected in the summer and autumn months compared to those harvested from St. Lawrence Island in the NBS in the spring. This regional distinction aligns with sea ice organic carbon levels measured in walrus prey items (benthic macrofauna), in addition to the general distribution of HBIs deposited on the seafloor throughout the Pacific Arctic [76]. There was no significant difference between males and females in the Chukchi Sea suggesting similar foraging behaviors of the migratory animals during the summer and autumn. There were proportionally greater contributions of sea ice organic carbon in the diet of females compared to males in the NBS for each year of this study, which may be attributed to females walruses seeking out lipid-rich prey items of a higher quality, differing foraging behavior (i.e. reduced foraging by males at this time of the year) and/or a requirement for elevated lipid stores. Sea ice organic carbon contributions were similar between the NBS and Chukchi Sea walrus diets in 2012, yet differed in 2014. Sea ice persistence was above average for the NBS in 2012 but below average in the Chukchi Sea, which was also the current record low summer sea ice extent for the Arctic Ocean as a whole. This result theoretically supports the connection between trophic transfer of HBIs and sea ice conditions, but, a comparison of additional years from both regions may be needed to validate this tentative finding. Preliminary data suggest that walrus calves may have elevated sea ice organic carbon in their diets, perhaps driven by an enhanced requirement for lipid-rich prey to support their growth and development, or regional influences owing to their time spent foraging in the Chukchi Sea. Additional research on sea ice organic carbon contributions in juvenile walrus diets and body condition metrics during low sea ice years would help resolve this uncertainty. There were limitations to this study owing to the opportunistic nature of the available samples resulting in uneven sample sizes between the Bering and Chukchi Seas. However, our data suggest that contributions of organic carbon derived from ice algae to the Pacific Arctic food web vary annually but there is likely differential assimilation between male and female Pacific walruses based on their foraging activities. Having demonstrated the proof of concept for this approach in walrus livers, coordinated sampling efforts in the future could be valuable for tracking the relationship between shifting carbon sources and Pacific walrus diets in response to declining seasonal sea ice. Overall, we conclude that ice algae provide proportionally more organic carbon than phytoplankton as the basal energy source for female and juvenile Pacific walruses, which may suggest an additional vulnerability for this species as seasonal sea ice continues to diminish in the Arctic.

## Supporting information

S1 Table Stable isotope data (carbon and nitrogen) and sea ice organic carbon (iPOC) data for the Bayesian model for estimating trophic positions. The clam species, *Macoma calcarea*, served as the benthic baseline. Sea ice organic carbon values for *M*. *calcarea* were taken from Koch et al. [41] and stable isotope values are from Kędra et al. [73] and McTigue and Dunton [72]. FG is the functional group variable.
(XLSX)

S2 Table Pacific walrus liver tissue sample collection data. Sample data includes location/region, year, month, sex, and age class of the specimen. Samples were assigned to either Distributed Biological Observatory (DBO) 1 or 4 for sea ice analysis purposes. The biomarker abundance data (IP_25,_ HBI II and HBI III) was used to calculate the H-Print and iPOC values. Monthly mean sea ice concentrations were collected from the assigned DBO region and the month indicated. Sea ice persistence values are reported by year.
(XLSX)
